# Use and Effectiveness of a Video- and Text-Driven Web-Based Computer-Tailored Intervention: Randomized Controlled Trial

**DOI:** 10.2196/jmir.4496

**Published:** 2015-09-25

**Authors:** Michel Jean Louis Walthouwer, Anke Oenema, Lilian Lechner, Hein de Vries

**Affiliations:** ^1^ Maastricht University Department of Health Promotion Maastricht Netherlands; ^2^ School for Public Health and Primary Care (CAPHRI) Maastricht University Maastricht Netherlands; ^3^ Open University of the Netherlands Department of Psychology Heerlen Netherlands

**Keywords:** intervention use, Web-based, computer tailoring, obesity, educational level, delivery strategy, matching

## Abstract

**Background:**

Many Web-based computer-tailored interventions are characterized by high dropout rates, which limit their potential impact.

**Objective:**

This study had 4 aims: (1) examining if the use of a Web-based computer-tailored obesity prevention intervention can be increased by using videos as the delivery format, (2) examining if the delivery of intervention content via participants’ preferred delivery format can increase intervention use, (3) examining if intervention effects are moderated by intervention use and matching or mismatching intervention delivery format preference, (4) and identifying which sociodemographic factors and intervention appreciation variables predict intervention use.

**Methods:**

Data were used from a randomized controlled study into the efficacy of a video and text version of a Web-based computer-tailored obesity prevention intervention consisting of a baseline measurement and a 6-month follow-up measurement. The intervention consisted of 6 weekly sessions and could be used for 3 months. ANCOVAs were conducted to assess differences in use between the video and text version and between participants allocated to a matching and mismatching intervention delivery format. Potential moderation by intervention use and matching/mismatching delivery format on self-reported body mass index (BMI), physical activity, and energy intake was examined using regression analyses with interaction terms. Finally, regression analysis was performed to assess determinants of intervention use.

**Results:**

In total, 1419 participants completed the baseline questionnaire (follow-up response=71.53%, 1015/1419). Intervention use declined rapidly over time; the first 2 intervention sessions were completed by approximately half of the participants and only 10.9% (104/956) of the study population completed all 6 sessions of the intervention. There were no significant differences in use between the video and text version. Intervention use was significantly higher among participants who were allocated to an intervention condition that matched their preferred intervention delivery format. There were no significant interaction terms for any of the outcome variables; a match and more intervention use did not result in better intervention effects. Participants with a high BMI and participants who felt involved and supported by the intervention were more likely to use the intervention more often.

**Conclusions:**

Video delivery of tailored feedback does not increase the use of Web-based computer-tailored interventions. However, intervention use can potentially be increased by delivering intervention content via participants’ preferred intervention delivery format and creating feelings of relatedness. Because more intervention use was not associated with better intervention outcomes, more research is needed to examine the optimum number of intervention sessions in terms of maximizing use and effects.

**Trial Registration:**

Nederlands Trial Register: NTR3501; http://www.trialregister.nl/trialreg/admin/rctview.asp?TC=3501 (Archived by WebCite at http://www.webcitation.org/6b2tsH8Pk)

## Introduction

Web-based computer-tailored interventions are increasingly being tested to target health-related behaviors, such as physical activity, dietary intake, and smoking [1,2]. These interventions are potentially cost-effective because they can reach many people with individualized information via the Internet for relatively low costs [1,3-5]. Unfortunately, these interventions often are not optimally used by the intended target groups. A rapid decline in use of intervention sessions in the first weeks after initial participation is seen, in particular among people with a low educational level [6-13]. As a result, many people will not be exposed to all essential intervention content if it is provided in multiple sessions over a longer period of time. This limits the potential impact of such interventions because evidence suggests that repeated intervention use is necessary to achieve sustainable behavioral changes [13-19]. Hence, many scholars have highlighted the necessity to increase research on strategies that can improve the (prolonged) use of Web-based computer-tailored interventions, particularly among people with a low educational level [6,11,20-22]. However, although research into this area is growing, thus far studies have yielded no or only modest improvements in intervention use [7,11].

Hence, the main aim of this study was to contribute to the required insight into how the use of Web-based computer-tailored interventions can be improved. This study is part of a randomized controlled trial (RCT) that has demonstrated that a video version of a Web-based computer-tailored obesity prevention intervention is more effective in reducing body mass index (BMI) and energy intake than a similar text version [23]. The first study aim was to examine if the video version resulted in more intervention use compared to the text version among people with a low educational level in particular. To provide insight into other possible ways to increase intervention use, the second study aim was to examine if the delivery of intervention content via persons’ preferred intervention delivery format was associated with more intervention use. A related study aim was to examine if this match and more intervention use were related to better intervention effects. In addition, the final study aim was to examine demographic factors and intervention appreciation variables as potential predictors of intervention use.

Previous research has shown that there are multiple computer-mediated delivery modes that can be used to effectively communicate messages to people, such as text, video, and audio [24]. The idea to examine the additional effects of videos originated from the fact that most Web-based computer-tailored interventions merely consist of “dry” text-based information. Video-based messages are considered to be livelier and, therefore, more likely to be engaging and to stimulate revisits [25-32]. For example, a recent study has shown that providing video-tailored feedback can significantly increase the time spent with a Web-based physical activity intervention [30]. The use of videos, in particular, may be appropriate for persons with a low educational level because these individuals generally are less text-oriented [22,25,33]. In addition, matching the delivery format of intervention content with participants’ preferred intervention delivery format may also lead to prolonged intervention use and eventually better health outcomes [34-38]. According to the Elaboration Likelihood Model, an adequate match between a person’s preferences and educational materials will stimulate central route processing, which accordingly makes it more likely that positive changes are induced [39]. Conversely, a mismatch can reduce participation and may result in negative outcomes, such as dissatisfaction with the information and eventually poorer intervention effects [35,38].

The use of Web-based computer-tailored interventions can also be improved by gaining more insight into the factors that are predictive for intervention use. For example, prior research has suggested that interventions that are appreciated well are more likely to be used [21,40-42]. Based on Self-Determination Theory (SDT), it has been suggested that intervention use may be higher when an intervention is evaluated well on factors that can increase a person’s intrinsic motivation, such as perceived autonomy and relatedness [43,44]. Research has also demonstrated that intervention use is influenced by demographic factors; men and people with a low educational level are more likely to discontinue a Web-based computer-tailored intervention, whereas usage is higher among women and older persons [11,17,45]. More insight into the demographic characteristics predictive for intervention use offers the possibility to encourage revisits among people who are less likely to revisit a Web-based computer-tailored intervention [11].

In conclusion, the main aim of this study was to provide insight into how the use and, relatedly, the effectiveness of Web-based computer-tailored interventions can be improved.

## Methods

### Ethical Approval

The Ethical Committee of the Open University Heerlen reviewed the study protocol and decided that there was no objection to performing the study. The study is registered in the Dutch Trial Register (NTR3501).

### Study Design and Respondents

For this study, we used data from a RCT about the efficacy of the video and text version of the Web-based computer-tailored obesity prevention intervention. These 2 versions were compared to a waiting list control group. Baseline measurements (T0) took place between September 2012 and February 2013 and there was one follow-up measurement 6 months after baseline (T1). Participants were eligible to participate if they were at least 18 years of age, had a paid job, had a BMI between 18.5 and 30 kg/m^2^, and had sufficient command of the Dutch language. People with a physical condition that influenced their dietary or physical activity pattern (eg, diabetes) were excluded from participation.

Participants were recruited via occupational health centers, but mainly directly through worksites and via advertisements in newspapers. Participants had to register at the study website, where they could read more information about the study and the intervention. After registration, participants were randomly assigned to 1 of the 3 study conditions (video version, text version, control group) in a computer-determined sequence after which they received a username and password by email. With their account, they could log in to the website and fill out the baseline questionnaire. Participants in the intervention conditions were given access to the intervention 2 weeks after completion of the baseline questionnaire. To decrease the likelihood of attrition, participants received 2 email reminders per questionnaire. Participants could further win 1 of 100 cash prizes of €100 if they completed all questionnaires (ie, total amount of prizes was €10,000).

### Intervention

The video and text version of the Web-based computer-tailored intervention had the objective to prevent weight gain or achieve modest weight loss by guiding people in making and maintaining small changes in dietary intake and physical activity. Both versions had exactly the same content. In the video version, most educational content (ie, approximately 75%) was provided via videos, whereas the text version provided the educational content only via text without any visual elements. The text in the video version was used to give instructions about setting goals and making plans, delivering optional in-depth information (eg, about the small changes approach), and giving feedback about how to deal with many different barriers. In the videos, professional actors read the messages aloud by means of a news-driven format. The I-Change Model [46] and self-regulation theories [47,48] were used as the theoretical basis of the intervention. The intervention could be used for a maximum 3 months and consisted of 6 sessions, which each lasted approximately 15 minutes. Session 2 could be followed directly after session 1, but the subsequent sessions were weekly to monitor participants’ progress over time. To decrease the likelihood of attrition, participants received 2 email reminders per session. Detailed information about the development of the intervention can be found elsewhere [25].

The aim of session 1 was to help participants set an appropriate weight goal (ie, maintain current weight or lose a little weight) and a behavior change goal (ie, improve dietary intake, physical activity, or both). For this purpose, participants received tailored feedback about their BMI, dietary intake, physical activity level, and sociocognitive beliefs toward making changes in diet and physical activity (ie, attitude, self-efficacy, and social influence).

Session 2 aimed to help participants make appropriate “if-then” action plans (ie, implementation intentions) [49]. For this purpose, tailored feedback was given to indicate which specific behavior changes participants could make to achieve their weight goal. Subsequently, participants had to specify when, where, and how they were going to perform the desired behavior change. After this session, participants could start with the planned behavior change.

The last 4 sessions could be accessed in the next weeks, with at least 1 week between each session. The main aim of these sessions was to indicate whether or not participants had achieved their goals. Session 3, for example, provided tailored feedback about participants’ behavior change progress and offered the possibility to make coping plans. In addition, session 4 also consisted of narratives in which a role model told how his/her behavior change was going and how he/she dealt with difficult situations. In this session, participants could also change their goals and plans. Session 5 was similar to session 4, but also provided iterative feedback concerning participants’ success in attaining their weight goal. Finally, session 6 was similar to the previous session, but also offered the possibility to set a long-term weight goal and make plans for achieving this goal.

### Measurements

#### Outcome Variables

Intervention use was assessed by examining how many sessions were completed during the entire intervention period. Based on website tracking data, it was possible to assess whether or not participants had completed a particular intervention session. For each session, completion was scored as 1 and noncompletion as 0. These scores were summed, which resulted in a total score for intervention use ranging from 0 to 6 completed sessions.

Participants’ dietary intake was assessed at both T0 and T1 by means of a food frequency questionnaire consisting of 66 items. This questionnaire was based on a validated questionnaire concerning fat intake [50]. Our questionnaire mainly assessed intake of energy-dense products originating from 6 different food categories (ie, dairy products, sandwiches and fillings, food at dinner, sweet and savory snacks, hot and cold beverages, and alcohol). For each food product, the frequency (ie, number of days per week) and quantity (ie, servings per day) were assessed and, when applicable, portion size and type of product (eg, use of skimmed, semiskimmed, or whole milk) were assessed as well. A score for the average daily intake of calories from energy-dense food products was calculated by combining these questions with the energy value of each food product [51].

At T0 and T1, physical activity was assessed using the Short Questionnaire to Assess Health-Enhancing Physical Activity (SQUASH) [52]. Research has shown that this is a reliable and valid questionnaire to estimate the level of physical activity among Dutch adults [52]. Per category (ie, commuting activities, leisure time activities, household activities, and activities at work), participants had to indicate on how many days per week they engaged in this activity, the average time per day spent in doing this activity, and the intensity of the activity (ie, light, moderate, or vigorous). The scores on these questions were used to calculate a total score for the average daily minutes of moderate and vigorous intensity physical activity.

To assess BMI, participants were asked to report their height in meters and their body weight in kilograms as measured in the morning without clothes and shoes at both T0 and T1. In addition, participants who had not completed the online follow-up questionnaire at T1 were contacted by telephone to assess their body weight. In-line with the online questionnaire, these participants were asked to indicate their body weight in kilograms as measured in the morning without clothes and shoes.

#### Intervention Delivery Format Preference

At T0, intervention delivery format preference was measured by asking via which delivery format participants preferred to receive information in Web-based computer-tailored interventions (text only, videos only, combination of text and videos, or no preference). The answer to this question was combined with the assigned study condition to determine whether or not participants’ preference matched with the delivery format of the allocated intervention condition. This resulted in 2 groups of participants: (1) participants with a matched preference and (2) participants with a mismatched preference. Participants in the video condition were considered to have a match when they preferred to receive intervention content via a combination of videos and text (as the video version consisted of both video and text). Participants in the text condition had a match when they had a text-only delivery format preference. Participants in the video condition were considered to have a mismatch when they preferred to receive intervention content via video only or text only. Participants in the text condition had a mismatch when they had a delivery format preference for video only or a combination of video and text. It should be noted that participants who indicated that they did not have a preference for a particular intervention delivery format were not included in this variable (n=320). In addition, participants who were allocated to the control condition were also not included in this variable because they did not receive the video or text version of the intervention.

#### Appreciation of Intervention

At T1, appreciation of the intervention was assessed by means of 8 concepts. First, participants were asked to indicate on a 5-point Likert scale (1=low and 5=high) how they appreciated the information and feedback messages in the intervention: interesting, useful, understandable, and fitting to own situation. In addition, participants were also asked to give an overall rating of their impression of the intervention on a scale ranging from 1 (very poor) to 10 (excellent). Finally, participants’ perceptions of the intervention regarding autonomy, relatedness, and competence were assessed. These 3 concepts were derived from the SDT [43] and measured on a 5-point Likert scale (1=low and 5=high). First autonomy was assessed with 2 items by asking participants to which degree they experienced freedom in setting own goals and plans as well as in deciding which information they could read. Next, competence was assessed using 3 items. Participants had to indicate if the intervention had increased their confidence in their ability to manage their weight, dietary intake, and physical activity behavior. Finally, relatedness was assessed using 3 items by asking participants if they felt involved and supported by the intervention. A mean score was calculated for each of the 3 SDT concepts.

#### Demographics

Demographic characteristics were assessed at T0 and included gender (1=male; 2=female), age, and educational level (ie, the highest level of education completed). Educational level was classified into 3 categories: low (1=primary or basic vocational school), medium (2=secondary vocational school or high school), and high (3=higher vocational school or university) [53].

### Statistical Analyses

All statistical analyses were conducted using SPSS 20.0 (IBM Corp, Armonk, NY, USA), applying a significance level of .05 for single variables and .10 for interaction terms [54]. At both T0 and T1, multiple imputation was used to replace missing values on demographics, sociocognitive variables, and the outcome variables. Based on the dropout rate and the amount of missing values, the number of imputations was set at 40.

Descriptive statistics and frequencies were used to describe the demographic characteristics of the study population at baseline as well as use of the different intervention sessions. Potential differences between the 3 study conditions at baseline were examined using analyses of variance (ANOVA) with Tukey post hoc tests for continuous variables and chi-square tests with Bonferroni correction for categorical variables.

Difference in use between the video and text intervention was assessed using ANCOVA. Another ANCOVA was performed to examine differences in use between the video and text intervention per educational level. Difference in use between participants who were assigned to an intervention condition that matched or mismatched their preferred intervention delivery format was also assessed with an ANCOVA.

Linear regression analyses with interaction terms were performed to examine whether the intervention effects were moderated by (1) intervention use and (2) matching or mismatching intervention delivery format. Moderation of intervention use was examined by comparing the effects of the intervention conditions to the control condition. For matching, moderation was examined by comparing the effects of the 2 intervention conditions with one another. The effect analyses were conducted for each outcome variable separately (ie, BMI, dietary intake, physical activity). The regression analyses were further adjusted for potential confounders (eg, baseline behavior and baseline differences). In addition, all moderation analyses were performed with both the multiple imputation as a completers-only dataset.

Finally, a linear regression analysis with the enter method was carried out to assess which demographics and intervention appreciation variables predicted intervention use.

## Results

### Study Sample

**Figure 1:**
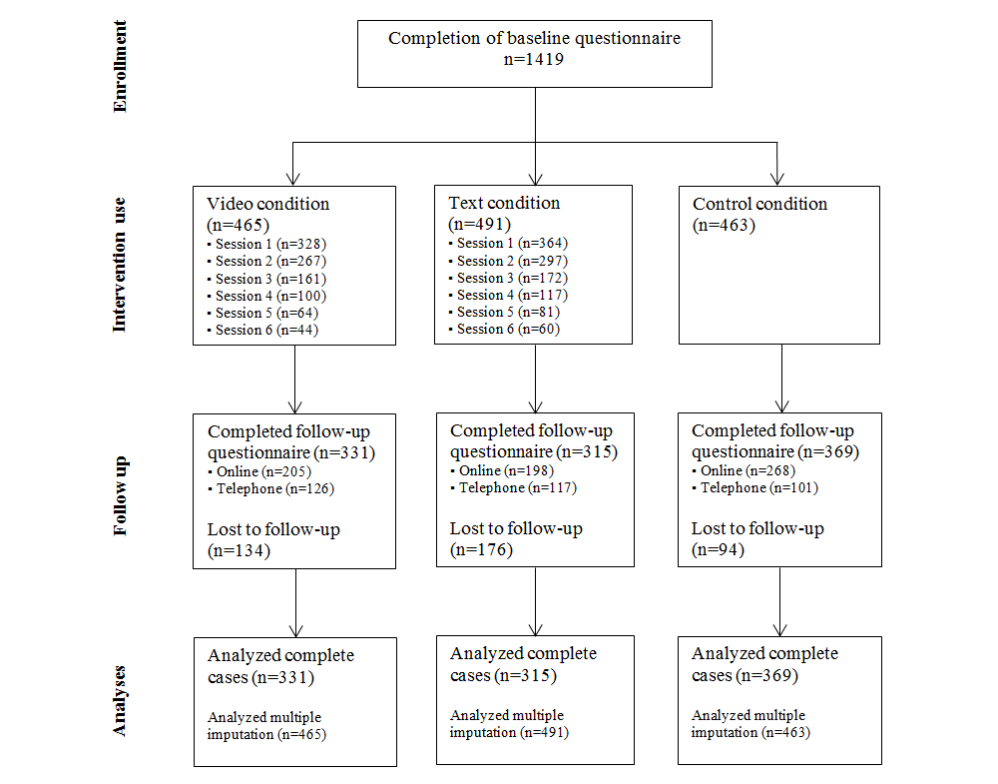
Flowchart of the enrollment, allocation, and participation of respondents.

The CONSORT-EHEALTH flowchart [55] shows the use of the intervention and participation throughout the study per study condition (see Figure 1). In total, 1419 participants completed the baseline questionnaire; at 6-month follow-up, data were collected for 1015 (71.53%) participants. Of the participants who completed the baseline questionnaire, only 328 of 465 (70.5%) participants in the video condition and 364 of 491 (74.1%) in the text condition also completed the first intervention session. Moreover, only 44 of 465 (9.5%) participants in the video condition and 60 of 491 (12.2%) participants in the text condition followed all intervention sessions. Overall, only 10.9% (N=956) of the participants completed all 6 sessions of the intervention.

Participants’ mean age was 48.12 (SD 11.52) years and 831 of 1419 (58.56%) participants were female (see Table 1). The distribution of educational level between the 3 study conditions differed significantly (χ^2^_4_=10.3, *P*=.004) at baseline (see Table 1). The number of participants with a low educational level was significantly higher in the control condition compared to the text condition. Moreover, the number of participants with a medium educational level was significantly higher in the text and control condition in comparison to the video condition. In addition, compared to the control condition, significantly more participants in the video condition had a high educational level.

**Table 1:** Characteristics of the study sample and differences between the study conditions.

Baseline characteristics			Full sample (N=1419)	Video (N=465)	Text (N=491)	Control (n=463)	*F* (*df1*,*df2*)	χ^2^ (*df*)	*P*
**Baseline**									
	Gender (female), n (%)		831 (58.56)	273 (58.7)	284 (57.8)	274 (59.2)		0.2 (2)	.91
	**Educational level, n (%)**							10.4 (4)	.004
		Low	214 (15.08)	75 (16.1)	67 (13.6)^a^	72 (15.6)^a^			
		Medium	436 (30.73)	118 (25.4)^a,b^	161 (32.8)^a^	157 (33.9)^b^			
		High	769 (54.19)	272 (58.5)^a^	263 (53.6)	234 (50.5)^a^			
	Age, mean (SD)		48.12 (11.52)	48.06 (12.05)	47.84 (11.58)	48.50 (10.92)	0.40 (2,2415)		.67
	BMI, mean (SD)		26.42 (2.33)	26.43 (2.25)	26.45 (2.37)	26.37 (2.38)	0.13 (2,2348)		.88
	Average daily minutes moderate and vigorous physical activity, mean (SD)		78.23 (83.40)	74.43 (73.27)	76.84 (81.11)	83.52 (94.51)	1.48 (2,2420)		.23
	Average daily energy intake, mean (SD)		1296.91 (501.04)	1308.36 (490.37)	1314.70 (497.42)	1266.51 (515.07)	1.33 (2,2378)		.27
	**Intervention delivery format preference, n (%)**							6.8 (4)	.34
		Text only	579 (40.83)	194 (41.7)	206 (42.0)	179 (38.7)			
		Video only	30 (2.12)	8 (1.7)	12 (2.4)	10 (2.2)			
		Combination video/text	489 (34.48)	162 (34.8)	175 (35.7)	152 (32.8)			
		No preference	320 (22.56)	101 (21.7)	97 (19.8)	122 (26.3)			
	**Matching intervention delivery format, n (%)**								
		Match	368 (48.61)	162 (44.5)^a^	206 (52.4)^a^	—		4.7 (2)	.03
		Mismatch	389 (51.39)	202 (55.5)^a^	187 (47.6)^a^	—			
**Follow-up**			(n=1015)	(n=331)	(n=315)	(n=369)			
	BMI, mean (SD)		25.99 (2.57)	25.87 (2.32)	25.96 (2.87)	26.12 (2.52)			
	Average daily minutes moderate and vigorous physical activity, mean (SD)		114.87 (109.68)	114.82 (100.55)	113.77 (97.46)	115.52 (120.48)			
	Average daily energy intake, mean (SD)		1078.97 (448.27)	1017.68 (434.46)	992.91 (460.40)	1157,55 (436.07)			

^a, b^ Values within a row with identical letters were significantly different as determined by chi-square tests with Bonferroni correction.

Most participants preferred to receive information in Web-based computer-tailored interventions via text only (40.80%, 579/1419) followed by a combination of video and text (34.46%, 489/1419). Only 30 of 1419 (2.11%) participants preferred to receive information via video only and 320 of 1419 (22.55%) participants had no preference regarding the delivery format. In total, 368 of 757 (48.6%) participants were assigned to an intervention condition that matched their preferred intervention delivery format. For example, 206 of 491 (52.4%) participants who were assigned to the text condition also preferred to receive information in Web-based computer-tailored interventions via text only. The distribution of matching and mismatching intervention delivery format between the 2 intervention conditions differed significantly (χ^2^_3_=4.7, *P*=.03). Significantly more participants in the text condition had a match compared to the video condition and vice versa. This result was also found when we included participants who had no preference for a particular intervention delivery format in the matching group (χ^2^_3_=8.3, *P*=.004).

### Intervention Use

The mean number of completed sessions was 2.07 (SD 1.91) in the video condition and 2.22 (SD 1.97) in the text condition, but this difference was not statistically significant (*F*_1,910_=1.55, *P*=.21). In the analyses stratified by level of education also, no significant differences were found in the average number of completed sessions between the text and video condition: low (*F*_1,127_=0.00, *P*=.84), medium (*F*_1,258_=2.47, *P*=.12), and high (*F*_1,503_=0.23, *P*=.63) educational level.

Yet, there was a significant difference in use between participants who were allocated to an intervention condition that matched their preferred intervention delivery format and those with a mismatch (*F*_1,910_=4.58, *P*=.03). The mean number of completed sessions was 2.24 (SD 1.96) among participants with a match, whereas the mean was 1.95 (SD 1.88) among those with a mismatch.

### Influence of Intervention Use and Matching/Mismatching Intervention Delivery Format

There were no significant video and text condition intervention use interaction terms for any of the outcome variables (see Table 2). This implies that intervention use did not have a moderating impact on the intervention outcomes. In addition, we also did not find significant condition intervention delivery format interaction terms for any of the outcome variables (see Table 2). This implies that the intervention outcomes were not influenced by whether or not participants were allocated to an intervention condition that matched their preferred intervention delivery format. These results were found with both the complete cases dataset as the multiple imputation dataset.

**Table 2:** Interactions terms regarding intervention use and matching/mismatching intervention delivery format for complete cases data.

Interaction terms^a,b^		β	*P*
**BMI**			
	Condition*match/mismatch	.024	.56
	Video condition*intervention use	–.090	.32
	Text condition*intervention use	–.084	.33
**Energy intake**			
	Condition*match/mismatch	.096	.29
	Video condition*intervention use	–.041	.27
	Text condition*intervention use	–.062	.10
**Physical activity**			
	Condition*match/mismatch	–.090	.33
	Video condition*intervention use	.022	.83
	Text condition*intervention use	–.023	.81

^a^ The moderation of intervention use was examined by comparing the intervention conditions to the control condition.

^b^ The moderation of match/mismatch was examined by comparing the intervention conditions with each other.

### Determinants of Intervention Use

The determinant analysis showed that participants with a higher BMI were significantly more likely to use the intervention more often (see Table 3). In addition, participants who felt involved and supported by the intervention (ie, feelings of relatedness) were also significantly more likely to use the intervention more often. The explained variance of the regression model was 16.0%.

**Table 3:** Determinants of intervention use (number of completed sessions) as assessed by multiple linear regression analysis.

Determinants		β	*P*
Study condition		.062	.37
Age		.090	.23
Gender		.133	.08
**Educational level**			
	Low vs medium	.012	.92
	Low vs high	.094	.45
BMI		.177	.02
Average daily minutes moderate and vigorous physical activity		.087	.24
Average daily energy intake		–.042	.56
The feedback messages fit to my own situation		–.082	.47
The feedback messages were understandable		.015	.88
The feedback messages were useful		.117	.27
The feedback messages were interesting		–.131	.24
Overall grade intervention (from 1 to 10)		–.082	.49
Feelings of autonomy		.148	.10
Feelings of relatedness		.291	.047
Feelings of competence		.018	.90

## Discussion

### Principal Findings

The main aim of this study was to examine how the use and effectiveness of Web-based computer-tailored interventions can be improved. For this purpose, we first examined if the use of a Web-based computer-tailored obesity prevention intervention can be increased by using videos as a delivery format. Secondly, we examined if the delivery of intervention content via participants’ preferred delivery format can increase intervention use. The third study aim was to examine if this match as well as more intervention use were related to better intervention effects. The final study aim was to identify which sociodemographic factors and intervention appreciation variables predict intervention use.

Intervention use (ie, number of completed intervention sessions) declined rapidly over time in both versions of the intervention. Contradicting our hypothesis, the video version was not used more often than the text version by the total study population or among participants with a low educational level. However, the intervention was used more often among participants who received intervention content via their preferred intervention delivery format. Our results further indicate that more intervention use and a matching intervention delivery format had not resulted in better intervention effects. In general, the intervention was more likely to be used more often by participants with a high BMI and participants who felt involved and supported by the intervention.

No support was found for the hypothesis that providing intervention content via (mainly) videos would result in more intervention use. This is an interesting finding because a previous study into the efficacy of this intervention has shown that the video version was appreciated significantly better than the text version [23]. Hence, a better appreciation does not necessarily lead to more intervention use. This suggestion should, however, be nuanced in-line with the fact that the appreciation of an intervention is also influenced by many other factors, such as the usability of an intervention and participants’ motivation to change [41]. In addition, our finding also contradicts a recent study that has shown that video-tailored feedback can result in more time spent on a Web-based computer-tailored physical activity intervention [30]. However, the findings of our study are in-line with 2 other studies that also concluded that the use of videos as delivery format has no effect on intervention adherence [22,56]. Hence, results thus far indicate that the use of videos may not be the most optimal solution to increase the use of Web-based computer-tailored interventions.

Our results further show that the use of these interventions can be slightly increased by delivering intervention content via users’ preferred intervention delivery format. Although videos did not increase intervention use, using this or another delivery format to match it with participants’ delivery format preference may increase intervention use. However, the potential of matching remains ambiguous because our study and a recent similar study has concluded that a matching intervention delivery format does not result in better intervention outcomes [56]. Therefore, future research should first provide a better indication about whether or not future interventions should offer participants a delivery format choice.

A possible explanation for the absence of moderation effects of intervention use and matching is the fact that the most important information relevant for achieving a successful behavior change was included in the first 2 intervention sessions. This information may have been sufficient to achieve behavior changes. Further, it has been suggested that the relationship between intervention use and health outcomes is curvilinear instead of linear, implying that there is a saturation point after which no further benefit will be obtained [57]. More is not always better and sometimes increasing requirements for participants can even have iatrogenic effects, such as lowered engagement [58]. However, there is also evidence that people need to be exposed to educational content multiple times before intervention effects can be expected [18,59-62]. For example, prolonged intervention use is necessary for learning and practicing skills over time. Hence, more research is needed to identify what the optimum number of intervention sessions is in terms of maximizing use and effects.

Overall, intervention use was low in both the video and text version. The steep decline in intervention use can possibly be explained by the fact that the intervention consisted of 6 information-rich sessions which required a high level of active involvement (eg, making plans and answering questions). For example, in session 2, participants had to answer approximately 25 questions and also make an action plan, which requires much cognitive effort. This probably was too demanding for participants and may have resulted in an overload and premature dropouts [11,41,63]. Another explanation could be the fact that the most important intervention content was included in the first 2 sessions. It is possible that the content of these sessions was sufficient for participants to enable them to change their behavior successfully. Hence, not all participants may have needed to use the last intervention sessions in which their behavior and weight goals were evaluated. These explanations are confirmed by previous research that has shown that people are primarily interested in a simple comparison of their behavior against the relevant guidelines. There is a lack of interest in behavior change counseling sessions that require a high level of active involvement [17,64]. As in all Web-based interventions, dropout can also be the cause of technical problems, such as errors on the website and slow video buffering [65-67]. For example, a study has shown that participants will quit an intervention when it takes more than 2 seconds to load a video, with each incremental delay of 1 second resulting in a 5.8% increase in dropout rate [67]. Finally, although the use of videos and tailoring can be considered sophisticated, recent technical developments, such as gamification and mHealth, may have raised users’ expectations of new products [68-71]. Hence, the video intervention possibly did not consist of sufficient innovative and attractive characteristics. In conclusion, these findings imply that still more research is needed into strategies that can increase the use of Web-based computer-tailored interventions.

Our determinant analysis shows that intervention use can possibly be increased by creating feelings of relatedness. Participants who felt involved and supported by the intervention (ie, feelings of relatedness) were more likely to use the intervention more often. According to the SDT, high feelings of relatedness will increase people’s intrinsic motivation to change and consequently make behavior changes more likely [43]. A recent study of the Web-based computer-tailored intervention has shown that the video version was evaluated significantly better on feelings of relatedness compared to the text version [23]. Hence, the use of videos is a potentially effective strategy to increase feeling of relatedness. Possibly, participants feel more involved and supported by a video delivery format because a person is actually talking to them in the videos and because it is easier to show empathy via spoken words.

### Strengths and Limitations

Our study is characterized by several limitations. The most important limitation of this study is the fact that intervention use was assessed by the number of completed sessions. Although it has been suggested that there is a high correlation between number of completed sessions and time spent on the intervention [12], this does not give any information about the exposure to and engagement with the intervention content. Other measures, such as use of specific pages and amount of information read, may give a better indication of actual intervention use [45,72]. Our measure for intervention use may not have been sensitive enough to find an effect of usage on the outcome measures. Hence, it is strongly recommended to include a more extensive measurement of intervention use in future studies examining Web-based computer-tailored interventions [7,21]. A second limitation concerns the measurement of preferred intervention delivery format. In contrast to directly asking participants for their preference, it has been suggested that it may be better to assess the preference strength on a scale ranging from low to high [73]. Third, it may have been better to examine the influence of matching/mismatching intervention delivery format by first stratifying for intervention delivery format preference before randomizing people to study conditions. Fourth, people with a low educational level were underrepresented in the study sample. However, this is a common finding in intervention studies because these people are difficult to recruit. In the statistical analyses, we have further corrected for this by including educational level as a covariate. Fifth, because of our applied randomization procedure participants were aware of the study condition to which they were allocated prior to completing the baseline questionnaire. This may have influenced participants’ responses to the baseline measurement.

Despite these limitations and the fact that we did not find support for all our hypotheses, this study provides a valuable contribution to the required research into this area. For example, an important strength is that we used a relatively new strategy (ie, use of tailored videos) to examine if the use of Web-based computer-tailored interventions can be improved among people with a low educational level in particular. Another strength is the fact that the analyses with the multiple imputation data resulted in exactly the same findings as the analyses with the complete cases data. In addition, we also corrected for potential confounding variables by including differences at baseline and predictors of attrition as covariates in the statistical analyses.

### Conclusions

The use of videos as delivery format of intervention content is not the solution to improve the use of Web-based computer-tailored interventions. Nevertheless, the use of these interventions can potentially be increased by providing intervention content via participants’ preferred intervention delivery format and ensuring that participants feel involved and supported by the intervention. The finding that more intervention use was not associated with better intervention outcomes implies that an intervention does not necessarily have to consist of many information-rich sessions. It may be sufficient to develop only 2 sessions that include the most important information necessary for achieving a successful behavior change. However, because only a few participants completed 3 or more sessions, more research is needed to identify what the optimum number of intervention sessions is in terms of maximizing use and effects. Until these strategies have been identified, it is recommended to minimize the number of sessions in future Web-based computer-tailored interventions and include the most important information for achieving a successful behavior change in the first sessions.

## Appendix

Multimedia Appendix 1 CONSORT-EHEALTH checklist V1.6.2 [55].

## Abbreviations

BMI: body mass index
RCT: randomized controlled trial
SDT: Self-Determination Theory
SQUASH: Short Questionnaire to Assess Health-Enhancing Physical Activity
