# Supplementary material for: Use and Effectiveness of a Video- and Text-Driven Web-Based Computer-Tailored Intervention: Randomized Controlled Trial
Source: J Med Internet Res. 2015 Sep 25;17(9):e222. doi: 10.2196/jmir.4496 (PMC4642388; doi:10.2196/jmir.4496)
Supplement: Multimedia Appendix 1 [file jmir_v17i9e222_app1.pdf]

CONSORT-EHEALTH Checklist V1.6.2 Report

(based on CONSORT-EHEALTH V1.6), available at [http://tinyurl.com/consort-ehealth-v1-6].

4496

Date completed

9/5/2014 8:27:31

by

Michel Walthouwer

Differences in effects and appreciation between a video and text web-based computer-tailored obesity prevention intervention: A randomized controlled trial

TITLE

1a-i) Identify the mode of delivery in the title

'video and text web-based computer-tailored obesity prevention intervention''

1a-ii) Non-web-based components or important co-interventions in title

'video and text''

1a-iii) Primary condition or target group in the title

'obesity prevention''

ABSTRACT

1b-i) Key features/functionalities/components of the intervention and comparator in the METHODS section of the ABSTRACT

'The intervention was developed using the Intervention Mapping protocol and based on self-regulation theories and the I-Change Model. The intervention consisted of six sessions, which each lasted about 15 minutes. In the video version, the core tailored information was provided by means of videos. In the text version the same tailored information was provided in text format.''

1b-ii) Level of human involvement in the METHODS section of the ABSTRACT

'web-based computer-tailored obesity prevention intervention''

1b-iii) Open vs. closed, web-based (self-assessment) vs. face-to-face assessments in the METHODS section of the ABSTRACT

'Outcome variables were self-reported and included BMI, physical activity, dietary intake, and appreciation of the intervention. Multiple imputation was used to replace missing values.''

1b-iv) RESULTS section in abstract must contain use data

'The baseline questionnaire was completed by 1419 participants and the 6 months follow-up measurement by 1015 participants (71.53%).''

1b-v) CONCLUSIONS/DISCUSSION in abstract for negative trials

Not applicable

INTRODUCTION

2a-i) Problem and the type of system/solution

'To examine if the use of videos can indeed improve the effectiveness and attractiveness, we developed two versions of a web-based computer-tailored intervention aimed to achieve small changes in dietary intake and physical activity in order to prevent weight gain among Dutch adults with a healthy weight or overweight (BMI between 18.5 and 30). Both versions had exactly the same content, but a different information delivery format.''

2a-ii) Scientific background, rationale: What is known about the (type of) system

'Web-based computer-tailored interventions meet all these requirements, since these interventions aim to change people's health behavior by providing individually adapted information via the Internet [14]. These interventions have the important advantage that they can be disseminated easily among a large target population against relatively low costs [11]. Research has already shown that web-based computer-tailored interventions can be cost-effective in the promotion of various health behaviors [15, 16]. Another advantage is that web-based interventions can be used in privacy and at a time and place participants find convenient. Moreover, people are increasingly using the Internet as source for searching health-related information [17-20], which further supports the use of a web-based intervention. Web-based computer-tailored interventions thus have the potential for a significant public health impact [21].''

METHODS

3a) CONSORT: Description of trial design (such as parallel, factorial) including allocation ratio

'We hypothesized that the video version would be more effective and appreciated better among lower educated participants.''

3b) CONSORT: Important changes to methods after trial commencement (such as eligibility criteria), with reasons

'Participants were initially recruited by handing out brochures during routine medical screenings by various occupational health centers. Unfortunately, this main recruitment strategy resulted in only few participants and additional recruitment strategies were necessary. Therefore, we also recruited participants directly through companies without interference of occupational health centers. An email was send to employees of the worksites that were willing to participate to inform them about the study. In addition, advertisements in national and local newspapers were also used to recruit participants.''

3b-i) Bug fixes, Downtimes, Content Changes

Not applicable

4a) CONSORT: Eligibility criteria for participants

'Criteria for participation were: being at least 18 years old, having a paid job, a BMI between 18.5 and 30, and sufficient command of the Dutch language. People with a physical condition that severely influenced their dietary or physical activity pattern (e.g. diabetes) were ineligible to participate.''

4a-i) Computer / Internet literacy

Not applicable

4a-ii) Open vs. closed, web-based vs. face-to-face assessments:

'Participants were recruited from September 2012 until February 2013. Participants were initially recruited by handing out brochures during routine medical screenings by various occupational health centers. Unfortunately, this main recruitment strategy resulted in only few participants and additional recruitment strategies were necessary. Therefore, we also recruited participants directly through companies without interference of occupational health centers. An email was send to employees of the worksites that were willing to participate to inform them about the study. In addition, advertisements in national and local newspapers were also used to recruit participants. All recruitment materials (i.e. brochures, emails, advertisements) included information about the intervention and the study as well as a hyperlink of the study website where participants could register to participate. After registration and giving online informed consent, participants were randomly assigned to one of the three study conditions in a computer determined sequence. After randomization they received a username and password by e-mail that gave them access to the study. Participants could subsequently fill out the online baseline questionnaire.''

4a-iii) Information giving during recruitment

'Participants were recruited from September 2012 until February 2013. Participants were initially recruited by handing out brochures during routine medical screenings by various occupational health centers. Unfortunately, this main recruitment strategy resulted in only few participants and additional recruitment strategies were necessary. Therefore, we also recruited participants directly through companies without interference of occupational health centers. An email was send to employees of the worksites that were willing to participate to inform them about the study. In addition, advertisements in national and local newspapers were also used to recruit participants. All recruitment materials (i.e. brochures, emails, advertisements) included information about the intervention and the study as well as a hyperlink of the study website where participants could register to participate. After registration and giving online informed consent, participants were randomly assigned to one of the three study conditions in a computer determined sequence. After randomization they received a username and password by e-mail that gave them access to the study. Participants could subsequently fill out the online baseline questionnaire."

4b) CONSORT: Settings and locations where the data were collected

Not applicable

4b-i) Report if outcomes were (self-)assessed through online questionnaires

'The outcome variables (i.e. BMI, dietary intake, and physical activity) were assessed using self-reports at both T0 and T1."

4b-ii) Report how institutional affiliations are displayed

Participants only knew that the study was carried out by Maastricht University.

5) CONSORT: Describe the interventions for each group with sufficient details to allow replication, including how and when they were actually administered

5-i) Mention names, credential, affiliations of the developers, sponsors, and owners

'The study was funded by ZonMw, the Netherlands Organization for Health Research and Development (grant number: 200110001)."
"Hein de Vries is the scientific director of Vision2Health, a company that licenses evidence-based innovative computer-tailored health communication tools. The other authors declare that they have no competing interests."

5-ii) Describe the history/development process

This information can be found in a related paper (study protocol).

5-iii) Revisions and updating

This information can be found in a related paper (study protocol).

5-iv) Quality assurance methods

Not applicable

5-v) Ensure replicability by publishing the source code, and/or providing screenshots/screen-capture video, and/or providing flowcharts of the algorithms used

This information can be found in a related paper (study protocol).

5-vi) Digital preservation

This information can be found in a related paper (study protocol).

5-vii) Access

'After randomization they received a username and password by e-mail that gave them access to the study. Participants could subsequently fill out the online baseline questionnaire. Two weeks after completion of this questionnaire, participants in the intervention conditions were given access to the intervention. Participants could use the assigned intervention for a maximum period of three months. Six months after baseline participants were asked by email to fill out the online follow-up questionnaire. When participants did not fill out this questionnaire, they received a reminder after one and two weeks."

5-viii) Mode of delivery, features/functionalities/components of the intervention and comparator, and the theoretical framework

'In the video version, the core tailored information was provided by means of videos. These videos had a news-driven format in which professional actors read the tailored information aloud, the same information that could be read in text in the text version of the intervention. It should further be noted that the video version also consisted of text-driven information, particularly for delivering the general non-tailored information."

5-ix) Describe use parameters

'The theoretical framework of the intervention was based on self-regulation theories [43, 44] and the I-Change Model [45]. The main determinants targeted with the intervention were knowledge, awareness, risk perception, attitude, social influence, self-efficacy, intention, skills, and action planning. Mainly the following behavior change methods were used to bring about change in these determinants: consciousness raising, tailored feedback on behavior and cognitions, goal setting, action and coping planning, and evaluation of goal pursuit. The intervention consisted of six sessions and each session lasted about 15 minutes."

5-x) Clarify the level of human involvement

Not applicable

5-xi) Report any prompts/reminders used

'When participants did not fill out this questionnaire, they received a reminder after one and two weeks. If the questionnaire was also not completed after these reminders, respondents were called to administer a short telephone questionnaire. To further decrease the likelihood of attrition, participants were informed that they could win one of hundred cash prizes of €100 if they completed all questionnaires [41]."

5-xii) Describe any co-interventions (incl. training/support)

Not applicable

6a) CONSORT: Completely defined pre-specified primary and secondary outcome measures, including how and when they were assessed

'Outcome variables
The outcome variables (i.e. BMI, dietary intake, and physical activity) were assessed using self-reports at both T0 and T1.
First, participants' body weight in kilograms and height in meters were assessed in order to calculate their BMI (weight/height²). To improve the adequacy of reporting, participants were asked to indicate their weight in the morning without clothes and shoes.
Dietary intake was assessed by means of a food frequency questionnaire consisting of 66 items, which was based on a validated questionnaire to assess fat intake [46]. The intake levels of mainly energy-dense products from six different food categories was assessed (i.e. dairy products, sandwiches and fillings, food at dinner, sweet and savory snacks, hot and cold beverages, and alcohol). For each food product, the frequency (i.e. number of days per week) and quantity (i.e. servings per day) were assessed. When applicable, type of product (e.g. use of skimmed, semi-skimmed, or whole milk) and portion size (e.g. size of candy bar) were assessed as well. These questions were combined with the energy value of each food product as indicated by the Dutch food composition table [47] in order to calculate a score for the average daily intake of calories from energy-dense food products.
Physical activity was assessed using the Short Questionnaire to Asses Health-Enhancing Physical Activity (SQUASH) [48]. This questionnaire has proven to be a reliable and valid tool to estimate the level of physical activity among Dutch adults [49]. The SQUASH takes about five minutes to complete and assesses participants' level of physical activity per category (i.e. commuting activities, leisure time activities, household activities, and activities at work). For each activity, participants had to indicate on how many days per week they engage in this activity, average time per day spent in doing this activity, and the intensity of the activity (light, moderate, or vigorous). By combining the frequency, duration, and intensity of physical activity, a total score was calculated for the average daily minutes of moderate-to-vigorous intensity physical activity.

Brief assessment of outcome measures
Participants who had not completed the follow-up questionnaire after several email reminders were contacted by telephone to conduct a brief assessment of the outcome measures. The telephone questionnaire consisted of 11 items concerning participants' body weight, dietary intake, and physical activity level. The purpose of the telephone questionnaire was to integrate it with the online follow-up questionnaire in order to replace missing values on the outcome variables. Participants' body weight was assessed by asking about their current weight in the morning without clothes and shoes. Unfortunately, analyses showed that for dietary intake and physical activity the online and telephone questionnaires resulted in totally different scores. Hence, the telephone questionnaire was not used for these variables.

Demographics
Demographic variables consisted of gender (1=male; 2=female), age, and educational level (i.e. the highest level of education completed) which was categorized into low (1=primary or basic vocational school), medium (2=secondary vocational school or high school), and high (3=higher vocational school or university) [50]. These variables were all assessed at T0 and used to describe the study population as well as to correct for potential confounding variables in the effect analyses.

Socio-cognitive variables
All socio-cognitive variables (i.e. self-efficacy, intention, and self-regulation skills) were assessed at T0 using adapted measures of previous studies [32, 51, 52] and a five-point Likert answering scale ranging from 1 (=low) to 5 (=high). These variables were assessed in order to correct for potential confounding variables in the effect analyses. A scale was computed by calculating a mean score.
Participants' self-efficacy was measured separately for physical activity (α=.83) and dietary intake (α=.81) using four items per behavior. Participants were, for example, asked about their confidence and ability to make changes to their diet and physical activity level.
Intention was measured with one item per behavioral outcome by asking participants if they intended to make changes to their diet and physical activity level within the next six months.
Self-regulation skills were measured for the types of skills that are important for successfully translating intentions into behavior change (i.e. goal setting, action planning, monitoring, and coping planning). Items were derived from existing instruments [53, 54]. Goal setting (α=.72) was measured with three items by asking participants if they set a goal in advance when they, for example, want to manage their weight. Next, action planning was measured with three items per behavioral outcome. Participants were asked if they had a clear plan when, where, and how they wanted to make changes to their diet (α=.90) and their physical activity level (α=.94). Monitoring (α=.74) was measured using four general items. Participants were, for example, asked if they monitored their weight and behavior on a regular basis. Finally, two items per behavioral outcome were used to assess coping planning. Participants were asked to which degree they were able to identify hindering situations in advance and thought that they were able to deal with these situations when making changes to their diet (α=.70) and physical activity level (α=.72).

Process evaluation
At T1, appreciation of the intervention was assessed by asking participants to which degree they thought the information and feedback in the intervention was interesting, useful, understandable, and fitted to their own situation. Next, participants were asked about their feelings of autonomy, relatedness, and competence during the intervention. These concepts were derived from the Self-Determination Theory [55] and the items were developed using existing questionnaires [26, 32, 51]. Autonomy was assessed by two items. Participants were asked if they had the feeling that they could decide by themselves which goals they could set and which information they could read in the intervention. Relatedness was assessed with three items by asking participants if they felt involved and supported by the intervention. Competence was assessed with three items by asking participants if the intervention had increased their confidence in their ability to manage their weight, dietary intake, and physical activity behavior. Furthermore, participants were asked to give an overall rating of the intervention on a scale ranging from 1 (=low) to 10 (=high). All other questions could be answered on a five-point Likert scale, ranging from 1 (=low) to 5 (=high). Finally, login-data was used to determine how many participants actually used the intervention."

6a-i) Online questionnaires: describe if they were validated for online use and apply CHERRIES items to describe how the questionnaires were designed/deployed

Not validated and CHERRIES not used

6a-ii) Describe whether and how "use" (including intensity of use/dosage) was defined/measured/monitored

'Finally, login-data was used to determine how many participants actually used the intervention."

6a-iii) Describe whether, how, and when qualitative feedback from participants was obtained

Not applicable

6b) CONSORT: Any changes to trial outcomes after the trial commenced, with reasons

No

7a) CONSORT: How sample size was determined

7a-i) Describe whether and how expected attrition was taken into account when calculating the sample size

'It was estimated that approximately 2000 participants were needed to complete the baseline questionnaire in order to be able to detect a medium sized effect (d=0.5) on BMI and behavior with a power of .90, a significance level of .05, and taking into account a drop-out percentage of 50%. This number of participants would also allow testing interaction effects between participants with a low, medium, and high level of education [31]."

7b) CONSORT: When applicable, explanation of any interim analyses and stopping guidelines

Not applicable

8a) CONSORT: Method used to generate the random allocation sequence

'participants were randomly assigned to one of the three study conditions in a computer determined sequence"

8b) CONSORT: Type of randomisation; details of any restriction (such as blocking and block size)

Not applicable

9) CONSORT: Mechanism used to implement the random allocation sequence (such as sequentially numbered containers), describing any steps taken to conceal the sequence until interventions were assigned

No

10) CONSORT: Who generated the random allocation sequence, who enrolled participants, and who assigned participants to interventions

The first author generated the random allocation sequence, enrolled participants, and assigned participants to interventions

11a) CONSORT: Blinding - If done, who was blinded after assignment to interventions (for example, participants, care providers, those assessing outcomes) and how

11a-i) Specify who was blinded, and who wasn't

Everyone, except the first research, was blinded after assignment.

11a-ii) Discuss e.g., whether participants knew which intervention was the "intervention of interest" and which one was the "comparator"

Participants knew to which study condition they were assigned.

11b) CONSORT: If relevant, description of the similarity of interventions

Not applicable

12a) CONSORT: Statistical methods used to compare groups for primary and secondary outcomes

'The effect analyses were conducted for each outcome variable separately (BMI, dietary intake, physical activity) using linear regression analyses with the enter method. These analyses were primarily conducted to examine the effects of the intervention conditions in comparison to the control condition, for which the study condition variable was recoded into two dummies (i.e. video versus control and text versus control). The analyses were adjusted for potential confounders (i.e. baseline behavior, predictors of attrition, and baseline differences). First, the analyses were run including study condition*educational level interaction terms in order to assess potential educational differences in intervention effects. When the interaction terms were significant, stratified analyses would be conducted. When interaction terms were not significant, the regression analyses were repeated without the interaction terms. Cohen's D effect sizes were calculated for all outcome variables [56]. As secondary analyses, we also compared the effects of the intervention conditions with each other. Moreover, complete case effect analyses were also performed in order to examine possible differences in results with the multiple imputation effect analyses.

Finally, the process evaluation data were analyzed. First, linear regression analyses with the enter method were performed including study condition*educational level interaction terms to identify potential educational differences in appreciation. When no interaction effects were found, independent samples t-tests were conducted to examine differences between the video and text condition on the process evaluation variables (i.e. appreciation).

All statistical analyses were conducted using SPSS 20.0, applying a significance level of 0.05 for single variables and 0.10 for interaction terms [57].''

12a-i) Imputation techniques to deal with attrition / missing values

'At both T0 and T1, multiple imputation was used to replace missing values on the socio-cognitive and outcome variables.''

12b) CONSORT: Methods for additional analyses, such as subgroup analyses and adjusted analyses

'First, the analyses were run including study condition*educational level interaction terms in order to assess potential educational differences in intervention effects. When the interaction terms were significant, stratified analyses would be conducted.''

RESULTS

13a) CONSORT: For each group, the numbers of participants who were randomly assigned, received intended treatment, and were analysed for the primary outcome

'In total, 1419 participants completed the baseline questionnaire, of which 465 belonged to the video condition, 491 to the text condition, and 463 to the control condition. At 6 month follow-up, data from 1015 (71.53%) participants was collected using the online and telephone questionnaires.''

13b) CONSORT: For each group, losses and exclusions after randomisation, together with reasons

See flow diagram

13b-i) Attrition diagram

See diagram.

14a) CONSORT: Dates defining the periods of recruitment and follow-up

'Participants were recruited from September 2012 until February 2013.''

14a-i) Indicate if critical "secular events" fell into the study period

Not applicable

14b) CONSORT: Why the trial ended or was stopped (early)

Not applicable

15) CONSORT: A table showing baseline demographic and clinical characteristics for each group

'Participants' mean age was 48.13 (SE 0.31) and 58.56% were female. The mean BMI was 26.42 (SE 0.06) and 73.50% of the study sample was overweight. The majority had a high level of education (54.19%), while fewest participants had a low educational level (15.08%).''

15-i) Report demographics associated with digital divide issues

Not applicable

16a) CONSORT: For each group, number of participants (denominator) included in each analysis and whether the analysis was by original assigned groups

16-i) Report multiple "denominators" and provide definitions

See flow diagram en table 1.

16-ii) Primary analysis should be intent-to-treat

'Moreover, complete case effect analyses were also performed in order to examine possible differences in results with the multiple imputation effect analyses.''

17a) CONSORT: For each primary and secondary outcome, results for each group, and the estimated effect size and its precision (such as 95% confidence interval)

See table 1, 2, and 3.

17a-i) Presentation of process outcomes such as metrics of use and intensity of use

See flow diagram and table 3.

17b) CONSORT: For binary outcomes, presentation of both absolute and relative effect sizes is recommended

Not applicable

18) CONSORT: Results of any other analyses performed, including subgroup analyses and adjusted analyses, distinguishing pre-specified from exploratory

'First, the analyses were run including study condition*educational level interaction terms in order to assess potential educational differences in intervention effects.''

18-i) Subgroup analysis of comparing only users

'First, the analyses were run including study condition*educational level interaction terms in order to assess potential educational differences in intervention effects.''

19) CONSORT: All important harms or unintended effects in each group

Not applicable

19-i) Include privacy breaches, technical problems

Not applicable

19-ii) Include qualitative feedback from participants or observations from staff/researchers

Not applicable

DISCUSSION

20) CONSORT: Trial limitations, addressing sources of potential bias, imprecision, multiplicity of analyses

20-i) Typical limitations in ehealth trials

'A limitation of this study is that all outcome measures were self-reported [64, 65]. We tried to gather objective data for BMI by working together with occupational health centers, but this recruitment strategy was unfortunately unsuccessful. However, research has shown that self-reported BMI does not affect results when used as continuous variable in a longitudinal study [66]. Moreover, although our drop-out rate is somewhat lower compared to previous research [26-29], it still is relatively high and implies that the attractiveness of the intervention deserves further attention. Possibly, when drop-out rates would have been lower and intervention use higher, the effect sizes would have been higher [30]. In addition, our sample size was lower as required by the power calculation and this has possibly ensured that no interaction effect of educational level could be found. Finally, we used a relatively short follow-up period of 6 months. Research with a longer follow-up period is necessary to examine whether or not the intervention effects will be maintained on the long-term.''

21) CONSORT: Generalisability (external validity, applicability) of the trial findings

21-i) Generalizability to other populations

'Based on our results, it can be concluded that a web-based computer-tailored obesity prevention intervention is most effective and attractive when it consists of video messages. Hence, to improve the impact of future web-based computer-tailored obesity prevention interventions, it is recommended to include videos as delivery format of health information. Our study shows that this is feasible and effective for Dutch adults with a healthy weight and limited overweight.''

21-ii) Discuss if there were elements in the RCT that would be different in a routine application setting

Not applicable

22) CONSORT: Interpretation consistent with results, balancing benefits and harms, and considering other relevant evidence

22-i) Restate study questions and summarize the answers suggested by the data, starting with primary outcomes and process outcomes (use)

'Our results show that there were no significant interaction effects, implying that the intervention effects were equal for all educational levels. The video version was the most effective intervention since it had a positive significant effect on BMI and dietary intake (compared to the control condition), while the text intervention only had a significant positive effect on dietary intake. No intervention effects on physical activity were found. Appreciation of the two intervention conditions did also not differ per educational level. Yet, the video version was appreciated better than the text version on several aspects of importance for intervention use. Overall, it can be concluded that the video intervention performed better than the text intervention regardless of people's educational level.''

22-ii) Highlight unanswered new questions, suggest future research

'However, further research with a longer follow-up period is needed to study how in particular people with a low educational level can be reached more effectively with these interventions.''

Other information

23) CONSORT: Registration number and name of trial registry

'Trial ID number: NTR3501''

24) CONSORT: Where the full trial protocol can be accessed, if available

Walthouwer, M. J., Oenema, A., Soetens, K., Lechner, L., & De Vries, H. (2013). Systematic development of a text-driven and a video-driven web-based computer-tailored obesity prevention intervention. BMC public health, 13(1), 978.

25) CONSORT: Sources of funding and other support (such as supply of drugs), role of funders

'The study was funded by ZonMw, the Netherlands Organization for Health Research and Development (grant number: 200110001).''

X26-i) Comment on ethics committee approval

'The Ethical Committee of the Open University Heerlen reviewed the study protocol and decided that there was no objection to performance of the study. The study is registered in the Dutch Trial Register (NTR3501).''

x26-ii) Outline informed consent procedures

'After registration and giving online informed consent, participants were randomly assigned to one of the three study conditions in a computer determined sequence.''

X26-iii) Safety and security procedures

'After randomization they received a username and password by e-mail that gave them access to the study.''

X27-i) State the relation of the study team towards the system being evaluated

'HdV is the scientific director of Vision2Health, a company that licenses evidence-based innovative computer-tailored health communication tools. The other authors declare that they have no competing interests.''
